# Supplementary material for: PGE2 Is Crucial for the Generation of FAST Whole- Tumor-Antigens Loaded Dendritic Cells Suitable for Immunotherapy in Glioblastoma
Source: Pharmaceutics. 2020 Mar 2;12(3):215. doi: 10.3390/pharmaceutics12030215 (PMC7150800; doi:10.3390/pharmaceutics12030215)
Supplement: Supplementary file 1 [file pharmaceutics-12-00215-s001.zip › pharmaceutics-721233-supplementary.pptx]

## Slide 1
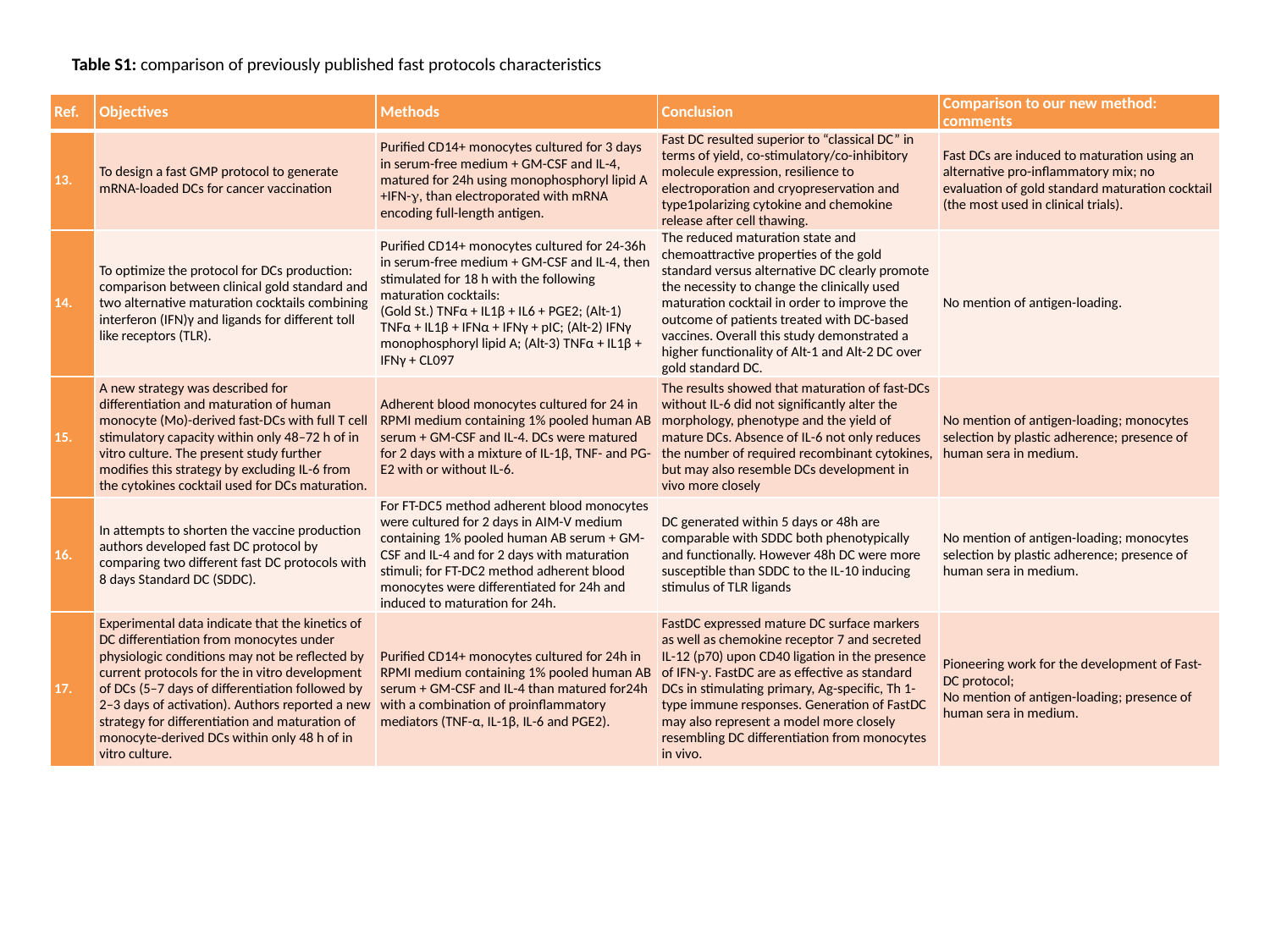

Table S1: comparison of previously published fast protocols characteristics
| Ref. | Objectives | Methods | Conclusion | Comparison to our new method: comments |
| --- | --- | --- | --- | --- |
| 13. | To design a fast GMP protocol to generate mRNA-loaded DCs for cancer vaccination | Purified CD14+ monocytes cultured for 3 days in serum-free medium + GM-CSF and IL-4, matured for 24h using monophosphoryl lipid A +IFN-γ, than electroporated with mRNA encoding full-length antigen. | Fast DC resulted superior to “classical DC” in terms of yield, co-stimulatory/co-inhibitory molecule expression, resilience to electroporation and cryopreservation and type1polarizing cytokine and chemokine release after cell thawing. | Fast DCs are induced to maturation using an alternative pro-inflammatory mix; no evaluation of gold standard maturation cocktail (the most used in clinical trials). |
| 14. | To optimize the protocol for DCs production: comparison between clinical gold standard and two alternative maturation cocktails combining interferon (IFN)γ and ligands for different toll like receptors (TLR). | Purified CD14+ monocytes cultured for 24-36h in serum-free medium + GM-CSF and IL-4, then stimulated for 18 h with the following maturation cocktails: (Gold St.) TNFα + IL1β + IL6 + PGE2; (Alt-1) TNFα + IL1β + IFNα + IFNγ + pIC; (Alt-2) IFNγ monophosphoryl lipid A; (Alt-3) TNFα + IL1β + IFNγ + CL097 | The reduced maturation state and chemoattractive properties of the gold standard versus alternative DC clearly promote the necessity to change the clinically used maturation cocktail in order to improve the outcome of patients treated with DC-based vaccines. Overall this study demonstrated a higher functionality of Alt-1 and Alt-2 DC over gold standard DC. | No mention of antigen-loading. |
| 15. | A new strategy was described for differentiation and maturation of human monocyte (Mo)-derived fast-DCs with full T cell stimulatory capacity within only 48–72 h of in vitro culture. The present study further modifies this strategy by excluding IL-6 from the cytokines cocktail used for DCs maturation. | Adherent blood monocytes cultured for 24 in RPMI medium containing 1% pooled human AB serum + GM-CSF and IL-4. DCs were matured for 2 days with a mixture of IL-1β, TNF- and PG-E2 with or without IL-6. | The results showed that maturation of fast-DCs without IL-6 did not significantly alter the morphology, phenotype and the yield of mature DCs. Absence of IL-6 not only reduces the number of required recombinant cytokines, but may also resemble DCs development in vivo more closely | No mention of antigen-loading; monocytes selection by plastic adherence; presence of human sera in medium. |
| 16. | In attempts to shorten the vaccine production authors developed fast DC protocol by comparing two different fast DC protocols with 8 days Standard DC (SDDC). | For FT-DC5 method adherent blood monocytes were cultured for 2 days in AIM-V medium containing 1% pooled human AB serum + GM-CSF and IL-4 and for 2 days with maturation stimuli; for FT-DC2 method adherent blood monocytes were differentiated for 24h and induced to maturation for 24h. | DC generated within 5 days or 48h are comparable with SDDC both phenotypically and functionally. However 48h DC were more susceptible than SDDC to the IL-10 inducing stimulus of TLR ligands | No mention of antigen-loading; monocytes selection by plastic adherence; presence of human sera in medium. |
| 17. | Experimental data indicate that the kinetics of DC differentiation from monocytes under physiologic conditions may not be reflected by current protocols for the in vitro development of DCs (5–7 days of differentiation followed by 2–3 days of activation). Authors reported a new strategy for differentiation and maturation of monocyte-derived DCs within only 48 h of in vitro culture. | Purified CD14+ monocytes cultured for 24h in RPMI medium containing 1% pooled human AB serum + GM-CSF and IL-4 than matured for24h with a combination of proinflammatory mediators (TNF-α, IL-1β, IL-6 and PGE2). | FastDC expressed mature DC surface markers as well as chemokine receptor 7 and secreted IL-12 (p70) upon CD40 ligation in the presence of IFN-γ. FastDC are as effective as standard DCs in stimulating primary, Ag-specific, Th 1-type immune responses. Generation of FastDC may also represent a model more closely resembling DC differentiation from monocytes in vivo. | Pioneering work for the development of Fast-DC protocol; No mention of antigen-loading; presence of human sera in medium. |
